# Synergistic Optimization of the Properties of Fiber-Content-Dependent PPS/PTFE/MoS_2_ Self-Lubricating Composites

**DOI:** 10.3390/polym18030410

**Published:** 2026-02-04

**Authors:** Zheng Wang, Shuangshuang Li, Liangshuo Zhao, Yingjie Qiao, Yan Wu, Zhijie Yan, Zhongtian Yin, Peng Wang, Xin Zhang, Xiaotian Bian, Lei Shi, Jiajie He, Shujing Yue, Zhaoding Yao

**Affiliations:** 1 School of Materials Science and Engineering, China University of Petroleum (East China), Qingdao 266580, China; wangzheng.-@163.com (Z.W.); yanzhijie@hrbeu.edu.cn (Z.Y.); yinxiaotian2025@163.com (Z.Y.); 20230026@upc.edu.cn (P.W.); 2 College of Chemistry and Chemical Engineering, China University of Petroleum (East China), Qingdao 266580, China; liwenjingli10@163.com; 3 College of Materials Science and Chemical Engineering, Harbin Engineering University, Harbin 150001, China; wuyan-007@hrbeu.edu.cn (Y.W.); 15945227533@163.com (X.Z.); bxt0615@hrbeu.edu.cn (X.B.); 4 CRRC Advanced Composites Co., Ltd., Qingdao 266041, China; csr_shil@126.com (L.S.); hejiajie8888@126.com (J.H.); yueshujing09136@163.com (S.Y.); syyao2008@126.com (Z.Y.)

**Keywords:** self-lubricating composites, CFRTP, friction performance, performance coordination optimization

## Abstract

This study systematically investigates the influence of short carbon-fiber (SCF) content on the mechanical, thermal, and tribological properties of self-lubricating polyphenylene sulfide (PPS) composites filled with PTFE and MoS2, addressing the critical need for high-wear resistance in Carbon-Fiber-Reinforced Thermoplastic (CFRTP) structural applications. The results identified 10 wt% SCF as the optimal content that achieved the best balance between load-bearing capacity and friction performance. The coefficient of friction μ and wear amount were reduced by 29.28% and 29.29%, respectively, compared to the PPS/PTFE/MoS2 composite material without SCF, and by 14.67% and 20.75%, respectively, compared to the material with excessive SCF filling (20 wt%). Finite-Element Analysis-Representative Volume Element (FEA-RVE) reveals the mechanism by which excessive content of SCF at the microscopic level leads to a slight decrease in mechanical properties. Critically, the tribological performance exhibited a discrepancy with bulk mechanical properties: above 15 wt% SCF, the wear rate worsened despite high mechanical strength, revealing that increased fiber agglomeration and micro-abrasion effects were the primary causes of performance deterioration. Further in-depth XPS analysis revealed a synergistic lubrication mechanism: In the optimal sample, an ultra-dense PTFE transfer film was formed to mask the underlying MoS2. This masking, coupled with the high surface activity of MoO3 particles leads to stronger physicochemical interactions with the polymer matrix, ensures the exceptional durability and stability of the tribo-film. This research establishes a complete structure–performance relationship by integrating mechanical, thermal, and tribo–chemical mechanisms, offering critical theoretical guidance for the design of next-generation high-performance self-lubricating CFRTPs.

## 1. Introduction

Carbon-fiber-reinforced thermoplastic (CFRTP) are increasingly adopted in aerospace and automotive industries due to their superior properties compared to carbon-fiber-reinforced thermosetting resins. CFRTP offers advantages including high fracture toughness at high voids, high productivity, long service-life, multi-level processability, and recyclability [1]. For instance, the Airbus A350 XWB employs CFRTP clamps manufactured through thermoforming to bond the outer skin to the frame, which forms part of the main structure [2]. Semi-crystalline resins like polyphenylene sulfide (PPS) and polyetheretherketone (PEEK) show greater promise in structural applications than amorphous resins due to their enhanced rigidity, strength, and heat resistance. However, PEEK’s high cost limits its large-scale application in composites, while polyamide (PA) with high moisture-absorption rates makes its composites unsuitable for prolonged service in humid environments [3]. In contrast, PPS exhibits excellent flexibility through its sulfur ether bonds, while its benzene rings increase rigidity. PPS also demonstrates outstanding thermal stability, extremely low moisture-absorption, and superior solvent resistance [4,5]. Therefore, CFRTP using PPS as the resin shows greater potential in certain application scenarios.

High-performance equipment such as a high-speed bearing retainer for aeroengine, ship stern shaft, and turbine machinery often operate under extreme conditions including rotational speeds of thousands-to-tens-of-thousands r/min, alternating contact stress, and extreme boundary lubrication or even dry friction [6]. In certain scenarios (e.g., inland navigation vessels), the use of lubricating oils is prohibited [7]. Traditional grease lubrication not only suffers from rapid centrifugal force-induced oil loss at high speeds but also risks contaminating precision components. This imposes integrated performance requirements on materials: “high strength, high heat resistance, and low friction wear.” However, Xu et al. [8] found that PPS/CF exhibits a relatively high intrinsic coefficient of friction (COF) (0.40 for dry sliding). Therefore, introducing solid self-lubricating components into the matrix to reduce friction and wear of PPS/CF under operational conditions is essential.

Polytetrafluoroethylene (PTFE), a classic solid lubricant, boasts advantages like high-temperature resistance and excellent chemical stability. Its tribological performance is primarily determined by the formation of a transfer film along the wear track, which reduces coefficient of frictions by preventing direct contact between friction pairs [9,10], making it the most widely used solid lubricant in industrial applications. Li et al. [11] filled PTFE into PEEK composites, while Zhang et al. [12] incorporated it into polyphenylene sulfone ether ketone (PPESK) composites. Both studies demonstrated significant reductions in coefficient of frictions and wear rates after filling. Shi et al. [13] found that simultaneously adding PTFE and multi-scale short carbon-fiber (SCF) substantially enhanced the tribological properties of PPS. The research indicates that the interlocking network structure formed by PTFE and SCF plays a crucial role in minimizing the coefficient of frictions and wear loss in PPS-based composites. However, Li et al. [14] revealed that excessive PTFE content not only reduces PPS’s coefficient of friction but also severely compromises its mechanical properties, compounded by PTFE’s inadequate lubrication durability under high loads [15,16]. This highlights that single-component additives (fillers or solid lubricants) cannot meet application demands, especially under harsh working conditions. Current research suggests that combining two or more solid lubricants with distinct mechanisms can significantly improve the tribological performance of polymer composites [17,18,19,20]. MoS_2_ crystals exhibit a hexagonal layered structure with extremely weak interlayer bonding (van der Waals forces), enabling interlayer sliding under external forces to reduce friction energy dissipation. As a non-reactive material, it neither chemically interacts with metal surfaces nor corrodes rubber materials, making it an excellent solid lubricant. Jiang et al. [21] demonstrated that introducing MoS_2_ as a secondary solid lubricant can compensate for PTFE’s inadequate lubrication durability under high-loads. The work of Hu et al. [22] revealed the synergistic effect of PTFE/MoS_2_ on the lubrication properties of polyimide-based CFRTP; however, no studies have explored the synergistic effect of PTFE/MoS_2_ on fiber-content-dependent PPS-based composites, and the performance improvements and underlying mechanisms of such composites remain unclear [23].

This study developed a PPS/PTFE/MoS_2_ ternary composite matrix incorporating short carbon-fiber (SCF) as the reinforcement phase [24]. Through systematic mechanical property tests (tensile, flexural, and impact), differential scanning calorimetry-thermogravimetry (DSC-TG), differential thermal gravimetry (DTG), fracture morphology observation, friction and wear experiments, and multidimensional surface characterization, combined with finite-element analysis (FEA) [25,26] using the Representative Volume Element (RVE) model for fiber-reinforced composites, we comprehensively investigated the SCF content-dependent structural and performance control mechanisms of the CFRTP. The research aims to meet the integrated requirements of “high strength, high heat resistance, and low friction/wear” for specialized materials under extreme operating conditions. This work provides experimental data and theoretical support for optimizing and engineering applications of self-lubricating CFRTPs in demanding transmission systems with medium-high loads, high rotational speeds, and boundary lubrication/dry friction coupling, demonstrating significant industrial value and scientific significance.

## 2. Experimental and Finite-Element Analysis

### 2.1. Materials and Chemical Composition

In this study, PPS/PTFE was used as the matrix, CF as the reinforcement phase, and MoS_2_ as the lubrication functional filler. The component content of the samples was designed as shown in Table 1. The PPS used was the Q250 model produced by China Binhua Group (Binzhou, China), CF uses the T800 model manufactured by Sinoma Shenying Carbon Fiber Co., Ltd. (Shanghai, China), with a tensile strength of 5490 MPa. The CF surface was thermoplastic modified using a polyamide coupling agent to increase the number of surface active functional groups, thereby enhancing the interfacial strength of the composite material [27]. The PTFE used is 5μ particle size produced by Zhejiang Wannengda Group Co., Ltd. (Quzhou, China), and the MoS_2_ was provided by Beijing Research Institute of Chemical Industry (Beijing, China), with a particle size of 10 μm in China. To systematically evaluate the performance, two different control groups were employed: Pure PPS was used as the baseline for mechanical tests to assess reinforcement efficiency, while PPS10P (PPS with 10 wt% PTFE) served as the primary benchmark for tribological evaluations to isolate the synergistic effects of MoS_2_ and SCF on friction and wear. For other material information, please refer to the Supplementary Materials.

### 2.2. Composite Fabrication

The composite material sample was fabricated via injection molding. First, PPS, PTFE, and MoS_2_ were heated and blended to form a mixture. The modified carbon-fiber (CF) was then impregnated into the molten mixture. Both the CF and the mixture were fed into a twin-screw extruder [28], where the CF was crushed at 300 rpm. The CF-containing (CF length is approximately 200–400 μm) extruded pellets are obtained by extrusion under pressure, and then are cut into 2 mm particles after cooling and air-drying. The particles are dried in a drying oven at 80 °C for 2 h, and then are injected into a mold preheated to 230 °C.

### 2.3. Mechanical Properties

Tensile and flexural tests were conducted using the LE-3000 universal mechanical testing machine manufactured by Shanghai Lishi Scientific Instrument Co., Ltd. (Shanghai, China) at a speed of 2 mm/min. Impact tests were performed with the XJUD-5.5 digital display cantilever beam impact tester produced by Chengde Kaosi Scientific Testing Co., Ltd. (Qingdao, China), featuring a 5 kg pendulum with a rated impact energy of 15 J. Three composite samples with varying fiber contents were tested, with the average value serving as the mechanical strength indicator for each composite material. During high-temperature (120 °C) tensile tests, the samples were preheated for 10 min prior to testing. The tensile-strength samples measured 150 mm × 20 mm × 5 mm, while the flexural- and impact-strength samples measured 80 mm × 10 mm × 5 mm. The fracture surfaces of the tested samples were analyzed using a APREO S LOVAC scanning electron microscope (SEM) manufactured by THERMO FISHER (Shanghai, China) [29,30].

In the friction and wear tests, the SFT-2M ball-disk type friction and wear tester was used. The small ball in the friction pair was selected as a Φ4 mm Si_3_N_4_ ceramic ball with a rotation radius of 3 mm. In the sliding friction test, the sliding speed was 0.6 m/s, and the load was 9 N. According to the Hertz Formula [31] for the contact between the ball and the plane in (1), the load was calculated as 378.2 MPa, and the friction time was 30 min. A surface observation and calculation of the specific wear rate were performed using a HYBRID C3 model 3CCD true color confocal microscope produced by Ogawa Seiki (Beijing) Trading Co., Ltd. (Beijing, China). The characterization and analysis of the friction surface and the mechanism of its friction behavior changes were conducted using an X-ray photoelectron spectrometer (XPS) produced by Thermo Fisher Scientific Inc. (Shanghai, China) and a APREO S LOVAC scanning electron microscope (SEM) manufactured by THERMO FISHER (Shanghai, China).

(1) $$\sigma_{\max}=0.388\sqrt[{3}]{PE^{2}\frac{1}{R^{2}}}$$

σ_max_ is the maximum contact pressure stress (MPa), P is the concentrated load (N), E is the elastic modulus (MPa), and R is the radius of the sphere (mm).

Each component underwent tribological and mechanical testing using three samples to avoid randomness. The friction curve was selected from the sample with uniform wear tracks. The wear test shall be conducted no less than three times. Mechanical performance testing standards use ASTM D3039 [24].

All binding energies were calibrated by referencing the adventitious C1s peak at 284.8 eV. High-resolution spectra were recorded with a pass energy of 30 eV and a step size of 0.1 eV to ensure sufficient energy resolution for peak deconvolution. Data processing and curve fitting were conducted using Avantage software (2023 Edition), employing a Shirley-type background subtraction.

### 2.4. Thermal Properties

The thermal stability test was performed using the JCT-1 comprehensive thermal analyzer manufactured by Beijing Hengjiu Experimental Equipment Co., Ltd (Beijing, China). The thermal properties of the designed material were analyzed through three methods: DSC, TG-DSC, and DTG [20,32,33]. The test was conducted at a heating rate of 15 °C/min, with a temperature range from room temperature to 800 °C, under a nitrogen atmosphere with a flow rate of 50 mL/min.

### 2.5. Multiscale Material Modeling

**Figure 1:**
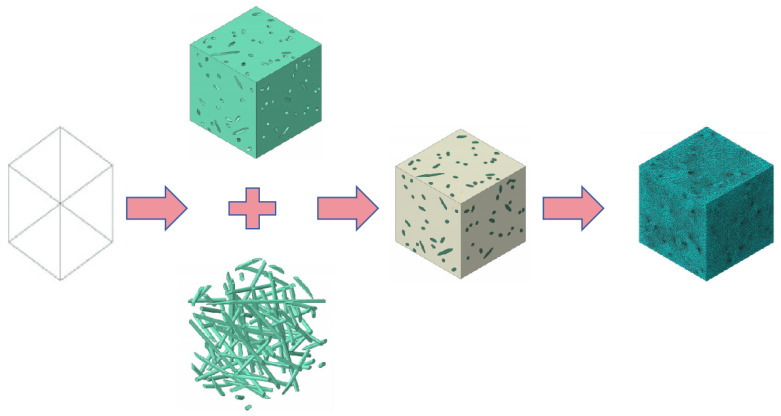
Generation of RVE of all the portions with composite model.

Digimat-FE can generate realistic RVE models suitable for the microstructure of various materials [34,35,36]. The homogenization method in Digimat-FE predicts the constitutive behavior of each phase in the pre-RVE model [37]. Stress distribution in the SCF reinforcement and matrix components of composites are difficult to analyze through experimental methods, but can be replicated and analyzed using finite-element modeling techniques. Therefore, in this study, Digimat-FE software (2025 Edition) was used to determine the microscale stress distribution in composites, to determine whether changes in mechanical strength are caused by inefficient stress-transfer between the matrix and SCF. The model analysis was completed in three steps. The first step defined pre-phase properties and microstructure, such as size, shape, position, clustering, orientation, and boundary conditions. The second step involved generating the RVE and creating the mesh. Subsequently, loads were applied under appropriate boundary conditions. To achieve better results, periodic boundary conditions were used in this study. The RVE analysis was completed in the final step; the final results were post-processed in probability distributions to provide uniform averages of the required properties. In the finite-element simulation, it was assumed that perfect bonding occurred between the SCF and matrix [38]. Figure 1 shows the RVE generated for all segments using the composite model.

## 3. Results and Discussion

### 3.1. Mechanical Property

**Figure 2:**
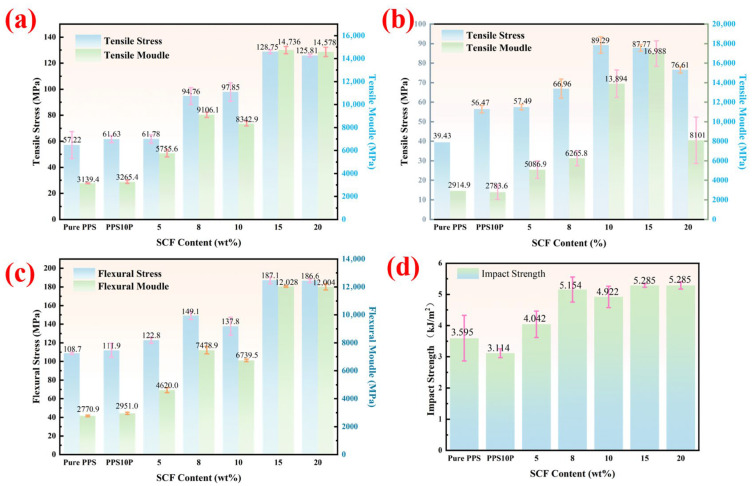
(**a**) Tensile performance at 25 °C; (**b**) tensile performance 120 °C; (**c**) bending performance at 25 °C; (**d**) impact performance at 25 °C.

**Figure 3:**
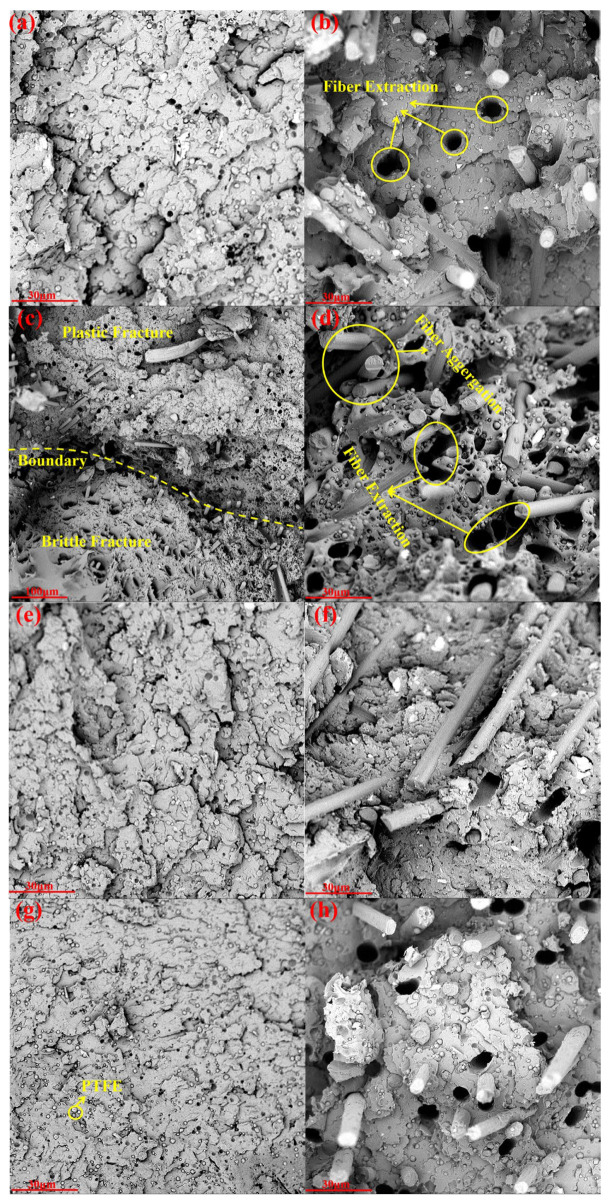
(**a**) PPS10P tensile fracture morphology at 25 °C; (**b**) C5 tensile fracture morphology at 25 °C; (**c**) C1 tensile fracture morphology at 120 °C; (**d**) C5 tensile fracture morphology at 120 °C; (**e**) PPS10P bending fracture morphology at 25 °C; (**f**) C5 bending fracture morphology at 25 °C; (**g**) PPS10P impact fracture morphology at 25 °C; (**h**) C5 impact fracture morphology at 25 °C.

Figure 2 displays the mechanical properties of the PPS/PTFE/MoS_2_-based composite, while Figure 3 shows its fracture morphology. The uniform distribution of PTFE within PPS is crucial for enhancing the composites’ tribological and mechanical performance. Results indicate that compared to pure PPS, the PPS/PTFE composite exhibits significantly deteriorated high-temperature tensile and impact properties. PPS molecular chains contain aromatic rings and sulfide bonds, whereas PTFE chains are fully fluorinated, resulting in weak interfacial bonding. As shown in Figure 3d, a microscopic gap exists between the PPS matrix and PTFE particles, forming a continuous “defect network”. Under stress, forces concentrate preferentially in these gaps. High-temperature tensile deformation further widens the gaps, preventing effective stress-transfer from the PPS matrix to PTFE particles through the interface. During impact, the interfacial gaps fail to effectively absorb energy, and PTFE’s mechanical properties are substantially inferior to PPS [39]. Table 2 presents the theoretical density, actual density, and porosity of the PPS/PTFE/MoS_2_ composite, with the actual density measured using the Archimedes displacement method [40].

**Figure 4:**
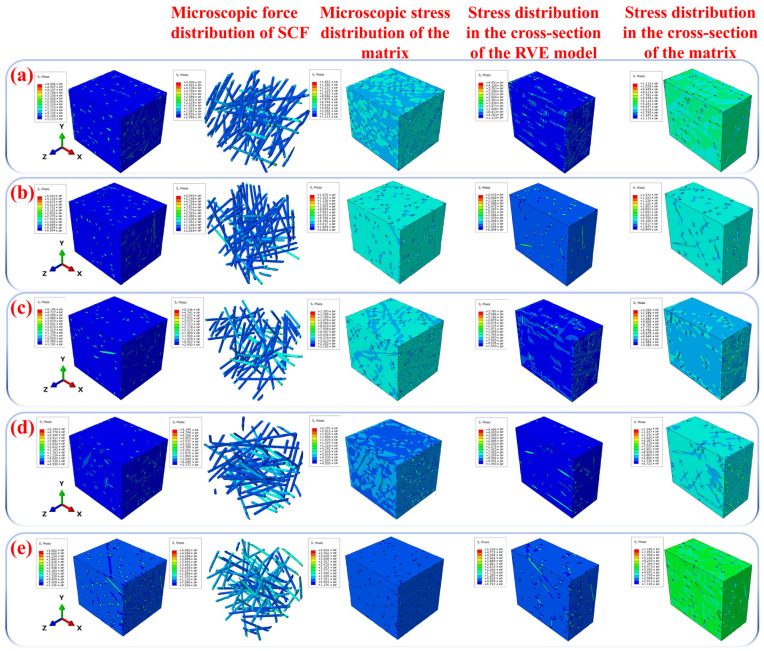
(**a**) Stress-distribution cloud map of C1’s RVE model after X-axis tension; (**b**) stress-distribution cloud map of C2’s RVE model after X-axis tension; (**c**) stress-distribution cloud map of C3’s RVE model after X-axis tension; (**d**) stress-distribution cloud map of C4’s RVE model after X-axis tension; (**e**) stress-distribution cloud map of C5’s RVE model after X-axis tension.

The mechanical properties of the composite material show an overall upward trend with increasing SCF content, but slightly decrease when exceeding 15 wt%. Compared to pure PPS, the composite with 15 wt% SCF exhibits a 125.0% increase in tensile strength and a 72.1% increase in flexural strength, along with 369.4% and 334.1% increases in tensile modulus and flexural modulus, respectively. Within the SCF content range of 5 wt% to 15 wt%, SCF acts as a high-load-bearing phase, effectively distributing external loads and reducing local stress concentration by alleviating the matrix’s burden. The high-modulus SCF also limits the matrix’s plastic deformation under stress, thereby enhancing overall rigidity. Additionally, SCF promotes PPS crystallization on its surface, forming a denser microstructure [2]. The fiber-bridging effect further inhibits crack propagation, improving fracture toughness. Figure 4 displays the stress-distribution cloud maps of RVE models for five C1–C5 samples under X-axis tensile loading. Results indicate that in C1–C4 models, SCF (short carbon-fiber) as the high-load-bearing phase primarily bears the load while reducing matrix stress. However, C5’s RVE model reveals significant stress concentration in its matrix compared to C1–C4, suggesting ineffective stress transfer from matrix to SCF. When the SCF content exceeds 20 wt%, the inter-fiber spacing becomes too narrow, which may lead to fiber aggregation and prevent the matrix from fully encapsulating the aggregated fibers. Table 2 shows that porosity increases by 29. 82% at 20 wt% SCF compared to 15 wt%, resulting in a sharp decline in the interfacial bonding strength between matrix and fibers. Consequently, loads are not effectively transferred to fibers, with more stress borne by the matrix around agglomerates. This causes localized matrix stress to directly exceed yield strength, ultimately leading to mechanical property degradation—a finding corroborated by experimental results. According to Auer et al. [41], the reduced tensile and flexural performance of 20 wt% SCF composites may also stem from excessive SCF interfering with PPS crystallization kinetics, restricting crystal growth space and forming distorted or incomplete crystal structures.

Although rigid SCF addition typically reduces the matrix ductility, the impact strength remains high (5.285 kJ/m^2^) under high fiber-loading (15–20 wt%). This is because the rigid SCF network acts as a weight-bearing skeleton, compensating for the weak interfacial defects introduced by the PTFE soft phase and effectively withstanding impact loads. As shown in Figure 3h, the impact-fracture surface features extensive fiber-extraction grooves, where friction generated during fiber extraction dissipates a significant portion of the impact energy. Additionally, the dense fiber network creates a bridging effect [42], which hinders crack propagation.

Figure 3 demonstrates that SCF-enhanced PPS/PTFE/MoS_2_ composites exhibit brittle-fracture modes in room-temperature tensile, impact, and flexural tests, primarily due to the brittle fracture of the PPS matrix and interfacial delamination of PTFE. However, Figure 3c,d reveal that at elevated temperatures, C1 exhibits both plastic and brittle fracture zones. This occurs because PPS softens and gains plastic-deformation capacity under high temperatures, while the weak interfacial bonding of PTFE still causes localized brittle-fracture. As SCF content increases, the matrix tearing induced by extensive fiber extraction gradually blurs the boundary between plastic and brittle fracture zones, ultimately transitioning to complete plastic deformation.

### 3.2. Thermal Performance Analysis

**Figure 5:**
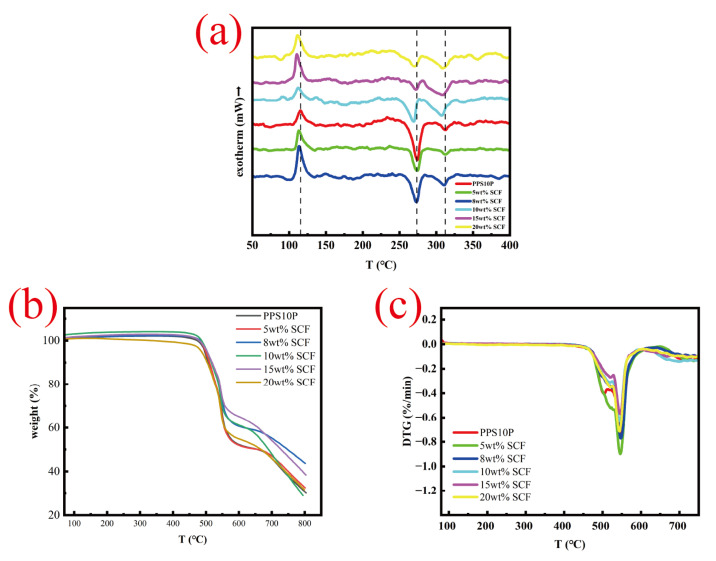
(**a**) DSC curve of composite materials; (**b**) TG curve of composite materials; (**c**) DTG curve of composite materials.

Figure 5a displays the temperature-dependent melting curves of PPS/PTFE composites with varying SCF contents. The curves show relative stability across the 50–100 °C low-temperature range. The pure PPS curve closely follows the SCF-containing composites, indicating no significant thermal transition within this temperature range and confirming the material’s predominantly solid state. Table 3 shows the hot blooded performance parameters.

A peak of exothermicity at 110 °C was observed, which was the cold crystallization peak of PPS. The cold crystallization was the characteristic of the polymer chain reorganization in the amorphous domain during the heating process, which reflected the crystallization ability or mobility of the polymer chain during the heat-treatment process.

At approximately 270 °C, the curve exhibits a distinct endothermic peak corresponding to the melting temperature of PPS, while PTFE’s melting peak occurs around 310 °C. As SCF content increases, the melting temperatures of both PPS and PTFE show a slight decrease. When SCF content reaches 10%, PPS’s melting-peak temperature drops to 267.8 °C, and PTFE’s melting temperature decreases to 307.4 °C. Subsequently, the melting temperatures of both materials exhibit a slight increase with further SCF addition. Meanwhile, the peak width and endothermic peak amplitude of PPS decrease with increasing carbon-fiber content, whereas those of PTFE increase, indicating that SCF significantly influences the thermal behavior during material melting.

As shown in Figure 5b,c, PPS10P and PPS composites with varying SCF content exhibit similar degradation behaviors, as evidenced by their identical TG curves. The thermal decomposition process generally progresses through three stages: (1) The weight loss is minimal at temperatures below 450 °C; (2) Exceeding 50% weight loss between 450 and 680 °C due to pyrolysis of PPS resin matrix into small molecules; (3) Residual carbon decomposition after 680 °C. PPS10P demonstrates a decomposition temperature (T_5%_) of 496.7 °C at 5 wt% weight loss, while SCF addition shows a significant upward trend in T_5%_ with a maximum increase of 5.2 °C. DTG images reveal the highest weight-loss rate (T_dTG_) at 550.2 °C. Thermal stability of the composites increases with SCF content before decreasing, with 8 wt% SCF exhibiting optimal thermal stability. This phenomenon results from the combined effects of the fiber-barrier effect [43] and pore-defect-induced effect [44]. As shown in Table 2, SCF content increases porosity from 0.70% to 3.57%. At lower SCF concentrations (5–8 wt%), porosity increases minimally. Rigid carbon-fibers effectively suppress thermal decomposition product diffusion and heat transfer, creating a labyrinth effect [45] that dominates thermal behavior. Consequently, SCF samples at 8 wt% demonstrate the highest thermal stability. However, at higher SCF concentrations (10–20 wt%), the porosity significantly increased to 3.57% due to elevated viscosity and fiber aggregation. The excess voids provided a larger free volume for the volatilization of degradation products. Moreover, the high-porosity-weakened-interfacial bonding, creating initiation sites for thermal degradation. During this phase, the detrimental effects of structural defects outweighed the barrier function of the fibers, resulting in reduced thermal stability for samples with SCF concentrations of 10 wt% to 20 wt%.

### 3.3. Friction Performance

**Figure 6:**
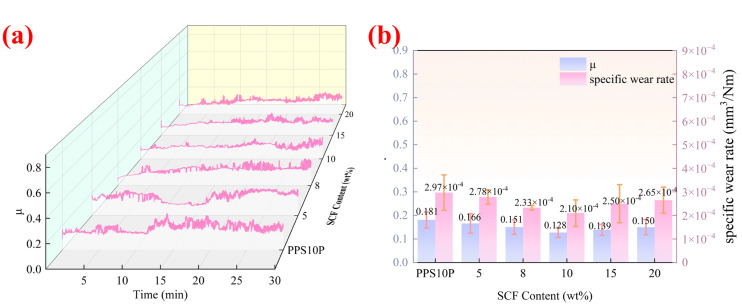
(**a**) Average coefficient of friction of PPS/PTFE/MoS_2_-based composites; (**b**) Specific wear-rate of PPS/PTFE/MoS_2_-based composites.

**Figure 7:**
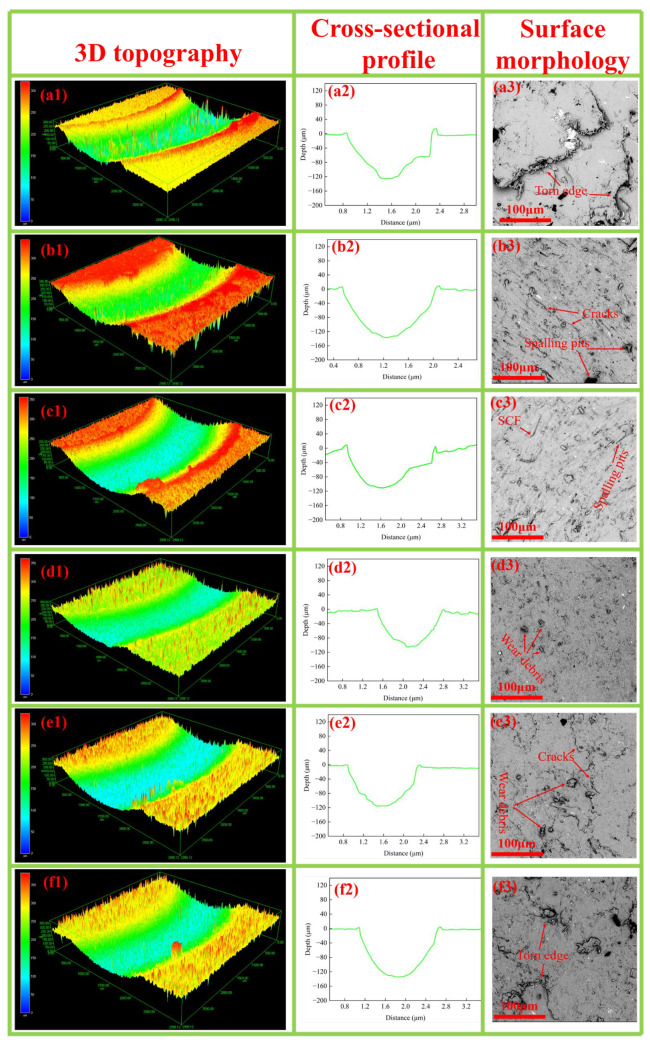
3D topographies, cross-sectional profiles and surface morphologies of wear tracks of the surface morphology: (**a1**–**a3**) PPS10P; (**b1**–**b3**) C1; (**c1**–**c3**) C2; (**d1**–**d3**) C3; (**e1**–**e3**) C4; (**f1**–**f3**) C5.

Figure 6 displays the average coefficient of friction and specific wear rate of this material system, with the specific wear rate [46] calculated using Equation (2). Figure 7 presents the surface friction marks of the material, characterized by 3CCD real-color confocal microscopy [47], alongside the SEM images of the friction surface morphology.

(2) $$K_{v}=\frac{V}{F\;\times \;S}$$

K_v_ is the volumetric specific wear rate [mm^3^/(N·m)], V is the wear volume (mm^3^), F is the load (N), and S is the total sliding distance (m). The wear volume V is calculated by multiplying the cross-sectional wear amount obtained from confocal topography measurement by the wear-ring circumference.

The average coefficient of friction was determined by calculating the arithmetic mean of the data recorded during the entire 30 min sliding test. Since the system reached a stable friction state rapidly, the values from the whole duration were representative of the steady-state performance.

As shown in Figure 6, while PTFE + MoS_2_ can form a slip film to reduce the coefficient of friction in pure matrix conditions, the absence of SCF reinforcement limits the matrix’s load-bearing capacity, resulting in relatively high wear-rates. Although the SCF addition disrupts partial continuity of the PTFE transfer film and increases micro-cutting forces, sacrificing some low-friction properties, it achieves a significant improvement in wear resistance. At low SCF concentrations, the reinforcement effect of SCF balances with the lubrication effect of PTFE + MoS_2_. The SCF-supported matrix reduces deformation, maintaining a complete and stable lubrication film where wear primarily occurs through “minor film migration”, leading to decreased wear-rates. However, when SCF content reaches 15 wt%, excessive aggregation dilutes the lubricating components, causing discontinuous lubrication films. Simultaneously, exposed SCF triggers abrasive wear, shifting the wear mechanism to “lubrication film failure + abrasive wear”, resulting in a marked increase in wear rates.

The surface morphology in Figure 7 reveals that without SCF (PPS10P), the material exhibits a rough, non-uniform flaky/blocky texture. The surface shows distinct protrusions and depressions, with localized shear-induced tearing edges formed by matrix compression. These characteristics indicate plastic deformation under friction when the thermoplastic composite matrix has a low load-bearing capacity, reflecting significant shear stress on the matrix. Micro-cracks and interfacial delamination traces demonstrate friction-induced bonding failure and matrix micro-cracking. Matrix plastic deformation tears the lubrication film, exposing PPS surfaces directly contacting Si_3_N_4_ ceramic balls, which triggers secondary friction, accelerates material spalling, and increases wear rates. When SCF is added, the surface morphology of friction marks shows more pronounced micro-cracks. At 10 wt% SCF content, the wear surface develops a relatively smooth yet rough micro-texture without large-scale tearing or deep pits, resembling a uniformly rough surface formed by wear. This indicates moderate friction exposure, with fine abrasive particles scattered around pits and on the surface—products of material shedding during friction. The results demonstrate that SCF at 10 wt% achieves dynamic equilibrium between its support function and the lubrication effect of PTFE+MoS_2_. With the addition of excess SCF (10–20 wt%), micro-cracks and tear-like edges gradually appear on the surface morphology. This microstructure is typical of lubrication failure under friction loads, where excessive SCF disrupts the continuity of the lubricating film. As SCF content increases, the C-O-C peak area expands, indicating oxidative degradation of the PPS matrix due to stress concentration and reduced interfacial adhesion. Under frictional shear forces, numerous SCF aggregates interpenetrate between PTFE/MoS_2_ layers, disrupting the continuous lubricating film distribution. These aggregates become crack sources and Three-body Abrasives, significantly exacerbating wear.

**Figure 8:**
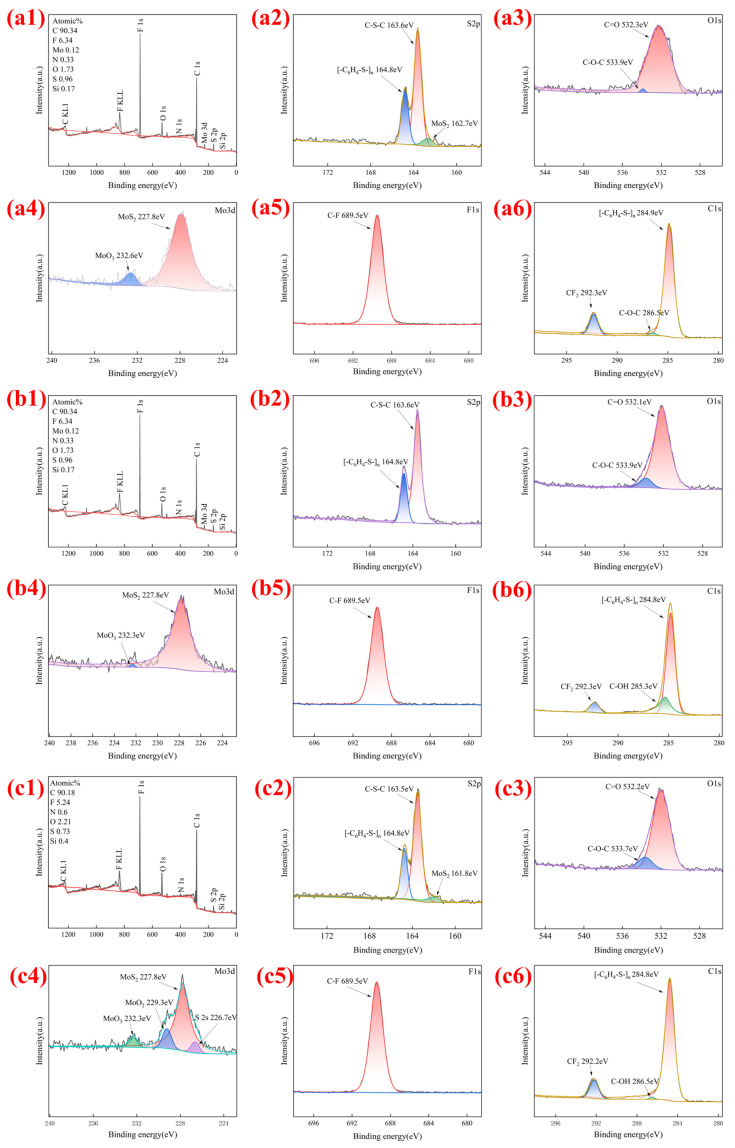
XPS spectrum analysis of friction area. (**a1**–**a6**) C1; (**b1**–**b6**) C2; (**c1**–**c6**) C3.

Figure 8 displays XPS energy spectrum analysis of samples with 5 wt%, 10 wt%, and 20 wt% SCF content at the wear scar. Analysis of Figure 8(a1,b1,c1) shows that at 10 wt% SCF, the N and Si elements in the friction pair balls are detected at the lowest levels (50% less than at 20 wt% SCF). This indicates that 10 wt% SCF forms a stable transfer film at the friction interface, significantly preventing direct contact between the composite material and friction pair balls. The minimized shear stress reduces N and Si element shedding from the friction pair balls, validating the trend where the coefficient of friction and specific wear-rate first decrease then increase with SCF content. Observations in Figure 8(a3,a6,b3,b6,c3,c6) reveal that the C-O-C peak area in the O1s spectrum increases with SCF content. After friction, 10 wt% and 20 wt% samples exhibit C-OH (hydroxyl) peaks in the C1s spectrum, indicating positive effects of friction oxidation. The generated C-OH and C-O-C polar groups form hydrogen bonds or chemical adsorption with mating surfaces, stabilizing high-quality transfer films. The 10 wt% sample with optimal lubrication performance shows the largest C-OH peak area, corresponding to the strongest chemical adsorption. This clearly demonstrates the transfer-film anchoring mechanism is induced by friction oxidation. Analysis of Figure 8(a2,b2,c2) reveals that MoS_2_ peaks appear in the S2p spectra at 5 wt% and 20 wt% concentrations, but the 10 wt% sample with optimal lubrication performance shows no MoS_2_ peaks. This indicates that in CFRTP composites with PTFE-MoS_2_ interactions, the PTFE transfer film forms on the outer layer of the MoS_2_ slip film and the MoS2 layer is covered. When SCF is present at 10 wt%, the composite forms a complete, dense, and continuous transfer film. However, at 5 wt%, the collective support strength is insufficient, causing the transfer film to crack under friction and expose underlying MoS_2_ layers. At 20 wt%, excessive fiber aggregates interpenetrate between PTFE/MoS_2_ layers, severely disrupting the transfer film’s continuity. Friction-induced micro-cutting further exposes and detects the underlying MoS_2_ layers. Analysis of Figure 8(a4,b4,c4) shows MoO_3_ peaks appearing in the Mo3d spectra, with significantly larger peak areas at 5 wt% and 20 wt% compared to 10 wt% samples. This is because MoS_2_ readily oxidizes to MoO_3_ under frictional heat [48,49]. In the 10 wt% sample, the friction temperature rise [50,51] was kept at a low level due to the moderate coefficient of friction and heat dissipation through carbon-fiber conduction. Meanwhile, the intact and continuous transfer film formed by the outer PTFE layer also protected MoS_2_, thereby inhibiting its conversion to the harmful MoO_3_ and preserving the lubricating phase of MoS_2_.

PTFE requires thermal softening through friction, shearing, and wire drawing to form a transfer film on the surface [52,53]. MoS_2_ readily undergoes partial oxidation under frictional heat, generating MoO_3_ [54]. As shown in Figure 8(a4,b4,c4), MoO_3_ peaks were detected in samples containing 5 wt%, 10 wt%, and 20 wt% MoS_2_ after friction. This indicates that MoS_2_ likely serves as the primary lubricant during the initial friction phase, protecting the substrate before the PTFE transfer film forms. Additionally, MoO_3_ exhibits high surface activity, making it prone to stronger physical or chemical interactions with PTFE or other polymer matrices [55], Therefore, it effectively reduces the shedding of the polymer matrix during the friction process. Although these MoO_3_ particles increase the sliding barrier, they are harder than MoS_2_. This means that the formation of MoO_3_ allows the matrix to withstand higher normal pressure and is less prone to lattice distortion.

**Figure 9:**
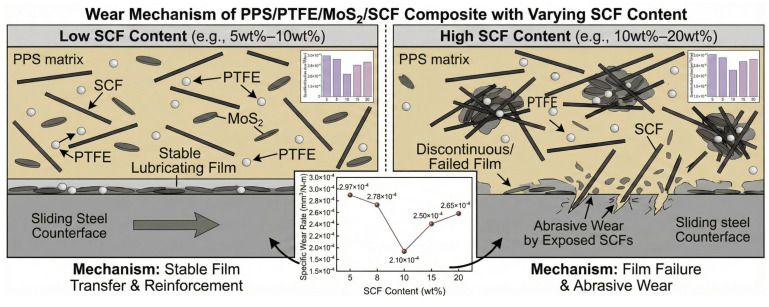
Mechanism of friction performance changes.

**Figure 10:**
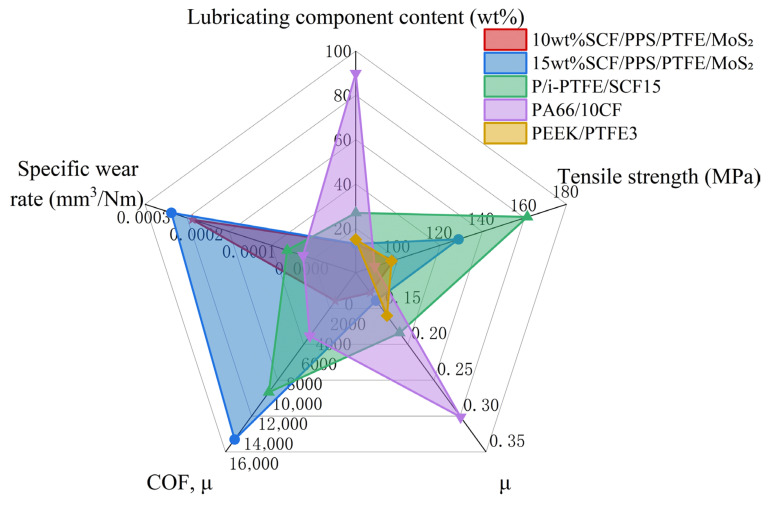
Comparison radar chart of performance of self-lubricating composite materials.

Figure 9 illustrates the friction performance variation mechanism of fiber-content-dependent thermoplastic composites with PPS/PTFE/MoS_2_ as the matrix. Figure 10 compares the performance of the SCF/PPS/PTFE/MoS_2_ system with other self-lubricating composites developed in recent years [56], it can be seen that the SCF/PPS/PTFE/MoS_2_ system demonstrates high lubrication performance on the basis of low filler content of lubricating components, while still maintaining high mechanical properties.

## 4. Conclusions

The effects of SCF content on the properties of self-lubricating PPS/PTFE/MoS_2_ composites were investigated systematically. The synergistic optimization mechanism of SCF content dependence was revealed from the perspectives of structure, mechanics, thermodynamics, and tribological chemistry.

1. Through comprehensive evaluation of the material’s mechanical, thermal, and tribological properties, we determined that 10 wt% SCF is the optimal ratio to maximize the overall performance of PPS/PTFE/MoS_2_ composites. The mechanical properties showed significant improvement, with the coefficient of friction (μ) and wear rate decreasing by 29.28% and 29.29%, respectively, compared to PPS/PTFE/MoS_2_ composites without SCF. These reductions were also 14.67% and 20.75% lower than those observed in materials with excessive SCF (20 wt%).
2. While 15 wt% SCF content achieves the highest macroscopic mechanical strength, its friction and wear performance begins to deteriorate. This reveals a mismatch between bulk mechanical properties and surface tribological characteristics. Higher SCF concentrations increase localized aggregate defects and amplify micro-cutting effects on surface fibers, disrupting the continuity of the surface transfer-film.
3. XPS analysis revealed a synergistic lubrication mechanism between PTFE and MoS_2_: In the 10 wt% sample with optimal lubrication performance, the characteristic peak of MoS_2_ disappeared. Simultaneously, the detection of MoO_3_ products in the transfer film indicated moderate frictional oxidation of MoS_2_. We hypothesize that MoS_2_ may act as the primary lubricant during the initial friction stage, the generated MoO_3_ particles have high surface activity and exhibit stronger physicochemical interactions with the polymer matrix, ensuring long-term stability with low friction and wear.

In conclusion, this study not only provides critical parameters for the precise formulation design of high-performance CFRTP self-lubricating composites, but more importantly, establishes a comprehensive theoretical framework to elucidate how SCF regulates the frictional behavior of polymer composites through the triple synergistic effects of structural, thermal, and chemical interactions. Future research should focus on enhancing macroscopic mechanical strength and reducing wear rates by modifying post-processing techniques, optimizing microstructure design, and adjusting filler dimensions, with the goal of achieving more durable ultra-low wear performance under high-load and high-speed operating conditions.

## Figures and Tables

**Table 1:** Content design of each component in the sample.

Number	PTFE (wt%)	MoS_2_ (wt%)	CF (wt%)	PPS
PPS10P	10	3	0	Bal.
C1	10	3	5	Bal.
C2	10	3	8	Bal.
C3	10	3	10	Bal.
C4	10	3	15	Bal.
C5	10	3	20	Bal.

**Table 2:** Actual density, theoretical density and voids fraction of composites at different wt.% of fiber content.

Number	Theoretical Density (g/cm^3^)	Actual Density (g/cm^3^)	Porosity (%)
PPS10P	1.436	1.426	0.70
C1	1.454	1.425	1.99
C2	1.465	1.428	2.53
C3	1.473	1.430	2.92
C4	1.492	1.451	2.75
C5	1.511	1.457	3.57

**Table 3:** Thermal stability of PPS/PTFE/MoS_2_—based composite materials.

Number	T_5%_ (℃)	T_50%_ (℃)	T_dTG_ (℃)
PPS10P	496.7	655.8	549.5
C1	497.5	641.7	547.2
C2	501.9	745.7	550.2
C3	497.9	684.8	544.6
C4	501.2	725.5	546.7
C5	485.2	666.0	545.7

## Data Availability

The original contributions presented in this study are included in the article/Supplementary Material. Further inquiries can be directed to the corresponding authors.

## Supplementary Materials

The following supporting information can be downloaded at https://www.mdpi.com/article/10.3390/polym18030410/s1, Table S1. Injection Molding Process Parameter Table; Table S2. Comparison table of SCF’s Mass fraction and Volume fraction; Table S3. FEA-RVE Modeling Details Table.
