# Supplementary material for: Synergistic Optimization of the Properties of Fiber-Content-Dependent PPS/PTFE/MoS2 Self-Lubricating Composites
Source: Polymers (Basel). 2026 Feb 4;18(3):410. doi: 10.3390/polym18030410 (PMC12899141; doi:10.3390/polym18030410)
Supplement: Supplementary file 1 [file polymers-18-00410-s001.zip › polymers-4124293-supplementary.pdf]

1.Raw material suppliers, specifications, and parameters

The PPS (polyphenylene sulfide) matrix resin uses the injection molding grade Q250 from Shandong Binhua Group Co., Ltd., China. Its melting temperature is 280-320°C. For the drying process, it is recommended to dry at 120-150°C for 3-4 hours, or at 150°C for at least 2 hours, with a dew point below -40°C. The moisture content should be less than 0.02%.

The PTFE (polytetrafluoroethylene) lubricant is produced by Zhejiang Wanan Da Group Co., Ltd., China, with a particle size distribution of 5 μm. Usually, no special pretreatment is required, but when dry mixed with SCF and resin, a small amount (0.3) of a coupling agent (γ-aminopropyltriethoxysilane) may be needed for pretreatment to improve interfacial compatibility.

MoS_2 (molybdenum disulfide) solid lubricant uses high-fineness powder with a particle size distribution of 10 μm from the Beijing Institute of Chemical Industry, China. To achieve better dispersion in a polymer matrix, the surface is organically treated with γ-aminopropyltriethoxysilane.

SCF (short-cut carbon fiber) uses the T800 model produced by Zhongfu Shenying in China, with a fiber diameter typically of 6-7 μm, tensile strength of 5490 MPa, tensile modulus of 294 GPa, elongation of 1.9%, and a density of 1.8 g/cm^3.

The coupling agent used is γ-aminopropyltriethoxysilane KH-550 from Hangzhou Horse Technology Co., Ltd., Zhejiang, China, with a density of 0.94-0.96 g/cm^3 at 25°C, a boiling point (760 mmHg) of 217°C, and a flash point above 96°C.

2.Preprocessing, Blending, and Pelletizing Process Parameters

PPS resin should be completely dried. SCF, PTFE, and MoS_2 can be used immediately after opening; otherwise, they need to be dried at 80-100°C for 1-2 hours to remove absorbed moisture.

Pre-coat PTFE and MoS_2 powders with a small amount of silane coupling agent (KH-550) in a high-speed mixer.

Melt blending was carried out using a co-rotating twin-screw extruder (L/D ≥ 48:1), with a feed zone temperature of 270℃, a melting/mixing zone temperature of 300℃, a die zone temperature of 300℃, and a screw speed of 300 rpm.

Feeding sequence: Add the dried PPS resin and the pre-mixed PTFE and MoS_2 powders together into the main feeder. After the melt is fully formed (around 2/3 of the screw), add SCF through the side feeder.

Vacuum degassing: Set up a vacuum vent before adding the fibers to remove volatile substances and moisture.

Residence time: Usually controlled at 60s to avoid excessive PPS degradation.

Granulation: Underwater pelletizing or strand water-cooled pelletizing. The pellet length is about 3mm. After granulation, the pellets are dried again at 120°C for 6 hours to ensure the final moisture content is less than 0.02%.

 https://doi.org/10.3390/polym18030410

3.Injection Molding Process Parameters

Table S1. Injection Molding Process Parameter Table.

Craft		Parameter
Barrel Temperature(℃)	Rear section	290
	Middle section	310
	front segment	320
	Nozzle	330
	Measured melt temperature	330
Mold Temperature(℃)		130-150
Injection molding stage speed（rmp）		60
Backpressure(MPa)		0.4
Injection Molding Pressure(MPa)		80
Pressure Holding	Time(s)	40
	Pressure(MPa)	50

4.FEA-RVE Modeling Details

Table S2. Comparison table of SCF's Mass fraction and Volume fraction.

Mass fraction(%)	Volume fraction(%)
5	5.94318
8	9.45278
10	11.7695
15	17.4824
20	23.0851

Table S3. FEA-RVE Modeling Details Table.

Modeling Details	Parameter
Inclusion	Cylinder
Aspect ratio	100
Orientation	Random 3D
Minimum relative distance between inclusions	0.05
Minimum relative volume (relative to elementary inclusion volume)	0.05
Element size definition	User defined
